# Pharmacognostic evaluation of *Artemisia maritima L.* a highly medicinal specie of genus *Artemisia*

**DOI:** 10.1016/j.sjbs.2022.103419

**Published:** 2022-08-17

**Authors:** Shah Zaman, Barkatulllah, Muhammad Zahoor, Syed Wadood Ali Shah, Zahid Ullah, Riaz Ullah, Amal Alotaibi

**Affiliations:** a Department of Botany, University of Malakand, Chakdara Dir Lower, 18800 KPK, Pakistan; b Department of Botany, Islamia College Peshawar, KPK, Pakistan; c Department of Biochemistry, University of Malakand Chakdara Dir Lower, 18800 KPK, Pakistan; d Department of Pharmacy, University of Malakand Chakdara Dir Lower, 18800 KPK, Pakistan; e Centre of Plant Science and Biodiverstiy, University of Swat, KPK, Pakistan; f Department of Pharmacognosy, College of Pharmacy, King Saud University, Riyadh 11451, Saudi Arabia; g Basic Science Department, College of Medicine, Princess Nourah bint Abdulrahman University, Riyadh 11671, Saudi Arabia

**Keywords:** Biological drug evaluation, Microscopic identification, Phytochemical characterization

## Abstract

The light and scanning electron microscopic observations were carried out for anatomical features of leaf, pollens and powder.Microscopic studies provide useful information for identification and authentication of adulteration in *A. maritima*. Nutritional analysis of *A. maritima* revealed that life fundamental macromolecules such as carbohydrates (49.63 %) crude proteins (13.17 %) and crude fibers (21.06 %) were present in sufficient quantity while crude fats (4.11 %) reported in low quantity. The life essential elements such as Mg (9.472 ± 0.011), Ca (4.152 ± 0.135) and Fe (4.112 ± 0.002) were found in high concentration while heavy metals reported under the safety threshold of WHO. These observations favored *A. maritima* an alternative of food.Appreciable quantity of phenolics (17.64 ± 0.574) and flavonoids (7.67 ± 0.069) were found while qualitatively active phytochemicals were reported.

The FTIR characterization of *A. maritima* crude powder revealed chromatogram in 3328.61 to 408.68 frequency range and 24 characteristic peaks on the basis of which different compounds of biological importance were classified. HPLC-UV technique quantifiedand identified six phenolic compounds morin,epigallocatechin gallate, catechin hydrate,ellagic acid, pyrogallol andrutin. Identification of compounds through GC–MS chromatogram revealed the presence of 46 compounds in methanolic fraction however 17 compounds of biological importance were selected.

*In-vitro* biological evaluation of *A. maritima* for antioxidant, antimicrobial, antidiabetic (12.61 ± 0.113 %) and cytotoxic activities (LC_50_ = 20 μg/ml) suggested that methanolic fractions exhibited the highest activity as compared to chloroform and ethyl acetate fractions. The MIC values of 10 or 15 mg/ml were recorded for most of the fungal pathogens. Antibacterial activity revealed 3.75 mg/ml of MIC values against *B. subtilis* and 1.87 mg/ml against *S. aureus, E. coli* and *P. aeruginosa*. *In-vivo* biological evaluation revealed thatmaximum inhibition was observed for crude extract at 250 mg/kg body weight. The mechanism underlined *in-vivo* analgesic responses was carried out which revealed that naloxone (morphine and tramadol antagonist) showed no prominent effect while Glibenclamide pretreatment minutely modified the analgesic action. These observations clearly indicted the absence of opiod receptors and involvement of ATP sensitive potassium channels.

## 1 Introduction

Among the angiosperm family Asteraceae is a largest family which includes over 20,000 species which are cosmopolitan in nature. Economically important genus *Artemisia* includes in family Asteraceae distributed along Europe, North America and Asia (Shehata et al., 2015). *Artemisia* is represented by well known 25 species in Pakistan (Zeb et al., 2019). *Artemisia* belongs to Anthemideae tribe (Nigam et al., 2019). The genus *Artemisia* of Anthemideae tribe has diverse biological and chemical contents and contains essential oil due to which current evaluations focused on phyto-constituent of this genus (Abad et al., 2012). Focused studies are required on *Artemisia* due to ecological, economic and species diversity (Vallès et al., 2003).

Decoction of *A. maritima* is important to cure intermittent fever (Ahmad et al., 2016). Leaf extraction of *A. maritima* is also used in Pakistan for skin diseases (Fahad and Bano, 2012). *Artemisia maritima* traditionally called “Zoon, Rooner and Tarkha” in the Northeastern area of Pakistan is used as anti-inflammatory, antimalarial and also antiseptic (Hayat et al., 2009). *Artemisia campestris* subsp. *maritima* have been evaluated for its antimicrobial, anti-inflammatory and anti-rheumatic activities (Behmanesh et al., 2007).

In the Sub-Continent of Indo-Pak the native communities and herbal industries generally face the difficulties in proper identification. They are made misguided and deal with completely different taxa (Khan et al., 2011).The raw materials used by the pharmaceutical industry and people are usually obtained from market, which may be contaminated, substituted or adulterated accidently or deliberately (Handa, 2004).The drug identification involves physical, chemical, biochemical and biological features (Alamgir, 2017). Present study designed to carry out various pharmacognostic features of *A. maritima* todifferentiate and authenticate it on the basis of various physical, chemical, biochemicals, and biological pharmacognostic features.

## 2 Materials and methods

### 2.1 Plant materials

Plant material shade dried after collected and was grounded using blender. An identified specimen was deposited to the herbarium of Islamia College Peshawar with voucher number ICP090618. Plant material of 2 kg was macerated in 10 L of methanol with occasional shaking was kept for one week. The extract was then filtered using Whatman filter paper No. 45. The filtrate obtained was evaporated in a rotary evaporator at 40 °C and about 50 g of methanolic crude extract was obtained. These processes were repeated twice. Crudemethanolic extract of (5 g) was stored in refrigerator at 2–4 °C for further use. The remaining extract was suspended with various organic solvents by their increasing polarity i.e. chloroform and ethyl acetate. All the fractions were dried using rotary evaporator. The dried fractions were stored at 4 °C in the refrigerator for future use (Jamil et al., 2012).

### 2.2 Chemicals and reagents

The chemical reagentsused inproximate analyses were NaOH, H_2_SO_4_, HNO_3_, C2SO and HClO_4_. Reagents used in Phytochemical analysis and characterization were FeCl_3,_ NH_3,_ CHCL_3,_ Na_2_CO_3_, NaNO_3_, C₆H₂(OH)_3_CO₂H, C_27_H_30_O_16_, KBr, column gradients system consists of solvent B (deionized water: methanol: acetic acid in the ratio of 180: 100: 20; v/v) and solvent C (water: methanol: acetic acid in the ratio of 80: 900: 20; v/v). Chemicals and reagents for *in-vitro* biological activities were (CH_3_)_2_SO, (DMSO), C_18_H_12_N_5_O_6_ (DPPH), C_6_H_8_O_6_ (Ascorbic acid), C_6_N_6_FeK_3_ (Potassium ferric cyanide), sabouraud dextrose agar (SDA), Turbinafine, Cefixime-USP, commercial sea salt, starch, Iodine, α-amylase, KI (potassium iodide), C_25_H_43_NO_18_ (Acarbose). Chemicals and reagents for *in-vivo* biological activities were CH_3_COOH (Acetic acid), CH_2_O (Formalin), C_16_H_25_NO_2_ (Tramadol), C_19_H_21_NO_4_ (Naloxone).

### 2.3 Microscopic characterization

Microscopic characterization such as light microscopy and scaning electron microscopy was carried out using standard procedure as adapted by Singh et al., 2018, Khan and Khan, 2020 using light microscope (Meiji MX5200H) and scanning electron microscope (JEOL-JSM 5910).

### 2.4 Proximate analysis (nutritional and elemental analysis)

Nutritional contents such as crude fat, crude protein, crude fiber and carbohydrate percentage were calculated by using method of Charles et al. (2005) as:

(1) $$Crude\;fat\;\left(\%\;of\;D.M\right)=\frac{initial\;weight\;-final\;weight}{Initial\;weight}\times 100$$

(2) $$\mathrm{Crude}\;protein\;\left(\%\right)=\frac{ml.\;of\;0.1\;N\;Sulphuric\;acid\;used\times 0.1\;(normality)\;\times 1.4007\times \;dilution\;factor\;\times \;F}{\mathrm{Weight}}$$

(3) $$Crude\;Fiber\;\left(\%\right)=\frac{W2-\;W3\;}{\;W1}\times 100$$

(4) Carbohydratecontent=100-(%moisture+%protein+%crudelipid+%crudefiber+%ash)

Determination of total ash (%), dry matter, moisture and gross food energy were calculated by Sadia et al. (2014) using the relation as given:

(5) $$Total\;Ash\;\left(\%\right)=\frac{Initial\;weight-Final\;weight}{Initial\;weight}\times 100$$

(6) Drymatter=100-%ofmoisture

(7) $$Moisture\;\left(\%\right)=\frac{\left(w2-w3\right)\times 100}{w2-w1}\times 100$$

(8) Grossfoodenergy(%)=[4×protein,4×carbohydrate,9×fat)kcal·100g-1

Elemental analysis was performed according to the standard procedure of Allen et al. (1974) with the help of Shimadzu AA-670 atomic absorption spectrophotometer using the relation:

(9) $$Nutrient\;cation\;in\;plants=\left(ppm\;in\;extract-blank\right)\times \frac{A}{W}\times dilution\;factor$$

### 2.5 Phytochemical screening (qualitative and quantitative)

Qualitative screening of tannins, saponin, flavonoid, terpenoid and alkaloid were carried using Khan et al. (2010). Quantitatively total phenolic contents (TPC) and total flavonoid contents (TFC) Sakanaka et al. (2005).

### 2.6 Phytochemical characterization

The Fourier Transform Infrared (FTIR) spectroscopy analysis was performed following Deshmukh and Ghanawat (2020). HPLC-UV characterization and quantification were carried out according to Zeb (2015). The chemical investigations through gas chromatography mass spectrometry (GC/MS) carried out according to Yaşar et al. (2005).

### 2.7 *In-vitro* biological evaluation

The DPPH free radical scavenging activity, total antioxidant capacity (TAQ) and total reducing power (TRP) was calculated according to Phull et al. (2017). In antifungal activity-seven fungal pathogenic strains e.g. *Aspergillus niger, Fusarium solani, Aspergillus fumigatus, Mucor specie, Helminthosporium solani, Candida albicans* and *Aspergillus flavus* were used. In antibacterial activity-four pathogenic strains were used including gram positive bacteria (*Staphylococcus aureus* and *Bacillus subtilis*) and gram negative (*Pseudomonas aeruginosa* and *Escherichia coli*) according to Jamil et al. (2012). Cytotoxic potential was carried out according to Meyer-Alber et al. (1992) by using the relation as:

(10) %Death=(Sample-control/control)×100

Determination of α-amylase inhibition by colorimetric assay as adapted by Funke & Melzig (2006) was used as:

(11) %enzymeinhibition=(ODx-ODy/ODz-ODy)×100

Whereas: ODx, ODy and ODz are absorbance of test samples, negative control and blank.

### 2.8 *In-vivo* biological evaluation

In-vivo biological analgesic activities such as writhing test by acetic acid and licking response by formalin according to Shoaib et al. (2019). Hot plate test was carried out according to Eidi et al. (2016).While mechanistic approach for the modulation of pain among all fractions and extracts carried out according to Muhammad et al. (2012).

## 3 Results

### 3.1 Microscopic characterization

a. **Foliar and pollen characterization**

**Fig. 1:**
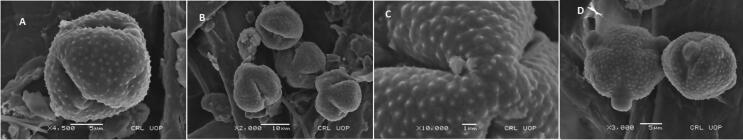
Scanning electron microscopy (SEM) of pollens of *A. maritima* (A = pollen pore, B = group of pollen with equatorial view, C = polar view and D pollen colpi).

The leaf and pollen anatomical features were studied out using light microscope and scanning electron microscope given in Table 1. Resultswere obtained in micro meter (µm) with ±SEM. The observations recorded are given in Fig. 1 and Fig. S1.

b. **Powder characterization**

**Table 1:** LM and SEM of leaf and pollen characters of *A. maritima.*

Foliar Characters (µm) ± SEM	Adaxial surface	Abaxial surface	Pollen Character (µm) ± SEM	Observations
Length of epidermal cell	11.4 ± 0.84	11.09 ± 1.74	Polar Diameter	3.3 ± 1.342
Width of epidermal cell	7.2 ± 0.72	5.98 ± 1.65		
No. of epidermal cells	53.4 ± 4.72	59.6 ± 7.98	Equatorial Diameter	5 ± 0.997
Length of guard cells	3.2 ± 0.38	2.58 ± 0.41		
Width of guard cells	2.1 ± 0.41	1.7 ± 0.44	P/E Ratio	0.67 ± 2.24
No. of stomata	13.4 ± 0.50	30.4 ± 8.11	Colpi Length	3.314 ± 1.4
Length of stomata	11.3 ± 0.63	9.7 ± 0.80		
Width of stomata	8.5 ± 0.84	7.98 ± 0.63	Colpi Width	2.56 ± 1.65
Length of subsidiary cell	12.8 ± 1.56	10.86 ± 1.91		
Width of subsidiary cells	6.6 ± 1.90	6.3 ± 1.35	Exine Thickness	1.17 ± 2.07
Length of stomatal pore	3.1 ± 0.36	2.68 ± 0.54		
Width of stomatal pore	2.1 ± 0.25	1.88 ± 0.43	No of fertile pollen	12.71 ± 5.50
Length of Trichome	N/A	11.38 ± 3.22		
Width of Trichome	N/A	3.24 ± 0.29	No of sterile pollen	5.143 ± 2.56
Stomatal Index	25.1 ± 5.8	51.1 ± 16.6		

Mean ± SEM (n = 7 number), LM = Light microscope and SEM = Scanning electron microscope.

Powder drug of *A. maritima* were evaluated for different characters (Table S1). Organoleptic evaluation revealed yellowish brown colour, aromatic odour and slightly salty taste. The observations under light and scanning electron are given in Fig. S2.

### 3.2 Proximate analysis

a. **Nutritional analysis**

The evaluation of *A. maritima* for nutritional analysis (in grams per 100 g dry weight) revealed good information for various nutritional components such ascrude fats, crude proteins, crude fibers, ash contents, moisture contents and carbohydrates (Fig. S3).

b. **Elemental analysis**

Elemental analysis of different elements (Fig. S4) such as calcium (Ca), magnesium (Mg), sodium (Na), potassium (K), iron (Fe), copper (Cu), zinc (Zn), manganese (Mn), Lead (Pb), cadmium (Cd) and chromium (Cr) were evaluated. The results revealed high concentration of Mg contents (9.47 ± 0.01) followed by Ca (4.15 ± 0.13) and Fe contents (4.11 ± 0.002). The current study showed that none of the heavy metals contents (Pb, Cd and Cr) reported in *A. maritima* exceeded the safety threshold.

### 3.3 Phytochemical analysis

Qualitatively and quantitatively various phytochemical were determined and results have been shown in Table S2.

### 3.4 Phytochemical characterization

#### 3.4.1 The Fourier Transform Infrared (FTIR) spectroscopy

TheFTIR chromatogramfrom the crude powder of *A. maritima* (Fig.S5) revealed 24 characteristic peaks and confirmed the presence of medicinally important functional groups (Table S3 identified from the literature) in the frequency range (3328.61 to 408.68).

#### 3.4.2 HPLC-UV characterization

The HPLC-UV chromatogram (Fig. S6) of methanolic extracts of *A. maritima* revealed identification and quantificationof six compounds (Table 2). Results revealed high concentration of Pyrogallol (1215.147 µg/ml) followed by morin (38.546 µg/ml).

**Table 2:** Identification and quantification of phenolic compounds in *A. maritima* extracts.

No. of Peak	Retention time (min)	Phenolic compounds Identity	HPLC-UV λ^max^ (nm)	Peak Area of sample	Peak Area of standard	Concentration (µg/ml)	Identification Reference
1	8	Epigallocatechin gallate	320	61.796	7261.47	0.085	Reference Standard
2	12	Morin	320	77.093	20	38.546	Reference Standard
3	16	Ellagic acid	320	271.117	319.24	8.492	Reference Standard
4	20	Catechin hydrate	320	22.426	78	2.875	Reference Standard
5	22	Rutin	320	48.514	2241.2	0.216	Reference Standard
6	28	Pyrogallol	320	123.216	1.014	1215.147	Reference Standard

#### 3.4.3 Gas chromatography mass spectrometry (GC/MS)

GC–MS chromatogram (Fig. S7) of *A. maritima* identified the presence of 46 compounds however 15 compounds of biological importance were selected (Table S4).

### 3.5 *In-vitro* biological evaluation

#### 3.5.1 Antioxidant activities

DPPH free radical scavenging activity, total reducing power assay (TRP) and total antioxidant capacity (TAC) were performed for different fractions of *A. maritima.*Antioxidant activities revealed that methanolic fractions exhibited the highest antioxidant activity (Table3).

**Table 3:** Ascorbic acid equivalent (% TRP, TAC and DPPH) antioxidant activity.

Fractions	Antioxidant assay		
	TRP	TAC	DPPH
Methanolic	66.7 ± 0.311^a^	44.02 ± 0.21^a^	80.7 ± 0.097^a^
Chloroform	54.4 ± 0.415^b^	37.35 ± 0.368^b^	79.087 ± 0.0489^b^
Ethyl acetate	39.24 ± 0.144^c^	16.43 ± 0.276^c^	73.1 ± 0.036^c^

**Key:** Values shown in the table are mean ± SE (n = 3). The means which share different superscript (a-c) letters in the columns are significantly (p < 0.05) different from each other.

#### 3.5.2 Antifungal activities

Antifungal activities of *A. maritima* crude methanolic extracts and their subsequent fractions (ethyl acetate and chloroform) were performed using agar tube dilution method at a concentration of 15 mg/ml (Table 4). The statistically variable results (at P < 0.05)revealedthat methanol and chloroform fractions were found effective.The most active fractions were evaluated for MIC value using disc diffusion method which revealed that most of the fungal strains were inhibited with MIC values of 10 or 15 mg/ml (Table S5).

**Table 4:** Antifungal activities of *A. maritima* (using agar tube dilution method).

	Fractions	Fungal strains						
		*A. niger*	*A. flavus*	*A. fumigatus*	*Mucor* sp.	*H. solani*	*C. albicans*	*F. solani*
		%Inh ± SE	%Inh ± SE	%Inh ± SE	%Inh ± SE	%Inh ± SE	%Inh ± SE	%Inh ± SE
Agar tube dilution method	Meth	44 ± 1.41^ab^	32.5 ± 2.12^a^	48 ± 1.41^c^	60 ± 1.41^a^	63.5 ± 3.53^c^	68 ± 1.41^bc^	59 ± 1.41^a^
	Chl	55.5 ± 2.12^c^	44 ± 2.82^b^	25 ± 4.24^a^	62 ± 2.82^a^	54.5 ± 6.36^ab^	71 ± 9.89^bc^	63.5 ± 0.70 ^a^
	E.a	45.5 ± 4.94^ab^	57 ± 1.41^c^	39.5 ± 3.53^b^	58.5 ± 2.12^a^	54 ± 1.41^ab^	53 ± 11.31^a^	63.5 ± 2.12 ^a^
	PC(+)	100 ± 0	100 ± 0	100 ± 0	100 ± 0	100 ± 0	100 ± 0	100 ± 0

**Key:** % Inh (percentage inhibition in mm), Meth (methanol), Chl (Chloroform), E.a (ethyl acetate), PC (positive control i.e. turbinafine).The means which share different superscript (a-c) letters in the columns are significantly (p < 0.05) different from each other.

#### 3.5.3 Antibacterial activities

Antibacterial assay was performed against the selected bacterial strains and six concentrations viz. 0.937 mg/ml, 1.875 mg/ml, 3.75 mg/ml, 7.5 mg/ml, 15 mg/ml and 30 mg/ml of each samples were used.The results (Table. 5) revealed that all the fractions were found to inhibit the growth of selected bacterial strains however methanolic fractions showed excellent activities at P < 0.05.Moreover, the active fractions were selected for MICs calculation the results are given in Table S6.

**Table 5:** Results of antibacterial activities (zone of inhibitionin mm) of different fractions of *A. maritima.*

Bacterial Strains		Gram positive bacteria						Gram negative bacteria					
		*Bacillus subtilis*			*Staphylococcus aureus*			*Escherichia coli*			*Pseudomonas aeruginosa*		
	Fractions	Conc. a	Conc. b	Conc. c	Conc. a	Conc. b	Conc. c	Conc. a	Conc. b	Conc. c	Conc. a	Conc. b	Conc. c
Disc diffusion method	Meth	19 ± 1.41^a^	17 ± 1.41^a^	15 ± 1.41^a^	18.5 ± 2.12^a^	15 ± 1.41^a^	10.5 ± 2.12^a^	18.5 ± 2.12^a^	15 ± 1.41^a^	10.5 ± 2.12^a^	18.5 ± 2.12^ab^	15 ± 1.41^a^	10.5 ± 2.12^a^
	CHl	14.5 ± 0.70^bc^	13.5 ± 0.70^b^	11 ± 0^b^	6.5 ± 0.70^c^	5.5 ± 0.70^c^	3.5 ± 2.12^c^	14.5 ± 0.70^bc^	10.5 ± 0.70^bc^	7 ± 1.41^bc^	19 ± 2.82^ab^	12 ± 1.41^bc^	6 ± 1.41^bc^
	E.a	13.5 ± 0.70^bc^	9.5 ± 0.70^c^	6.5 ± 0.70^c^	12 ± 1.41^b^	9.5 ± 0.70^b^	8.5 ± 0.70^b^	12.5 ± 0.70^bc^	9.5 ± 2.12^bc^	7 ± 2.82^bc^	15 ± 2.82^c^	10.8 ± 0.28^bc^	9 ± 1.41^bc^
	**PC1 + ve**	28.5 ± 0.70	26.5 ± 0.70	25.5 ± 0.70	28.5 ± 0.70	27 ± 0.70	28.4 ± 0.56	28.5 ± 0.70	27.35 ± 0.49	28.25 ± 0.35	27.95 ± 0.07	27.1 ± 0.14	27.87 ± 0.18
	**PC2 + ve**	19.5 ± 0.70	17.5 ± 0.70	18.5 ± 0.70	19.5 ± 0.70	18 ± 0.35	20.3 ± 0.42	19.2 ± 0.28	18.45 ± 0.63	19.35 ± 0.49	19.2 ± 0.28	18.36 ± 0.51	19.17 ± 0.24

**Key:** PC1 and PC2 (positive control i.e.Cefixime & Roxithromycin) Conc (concentration). Valuesshown in the table are mean ± SE (n = 3). The means which share different superscript letters (a-c) in the columns are significantly (p < 0.05) different from each other.

#### 3.5.4 Cytotoxic activity

The methanolic extracts of *A. maritima*showed significant cytotoxic activity at all concentration with LC_50_ value of 20 μg/ml (Table S7).

#### 3.5.5 Alpha-amylase anti-diabetic activity

*In-vitro* alpha-amylase enzyme inhibition activity of the extracts (Table S8) revealed highest enzyme inhibition (12.61 ± 0.113 %) for methanolic fractions.

### 3.6 *In-vivo* biological evaluation

Results from crude extracts and fractions revealed no mortality even at a maximum dose up to 1500 mg/kg (b.w) when orally administered. Hence, 125and 250 mg/kg dose for crude extracts and 75 mg/kg for subsequent fractions were chosen to evaluate analgesic activities.

#### 3.6.1 Writhing test by acetic acid

**Fig. 2:**
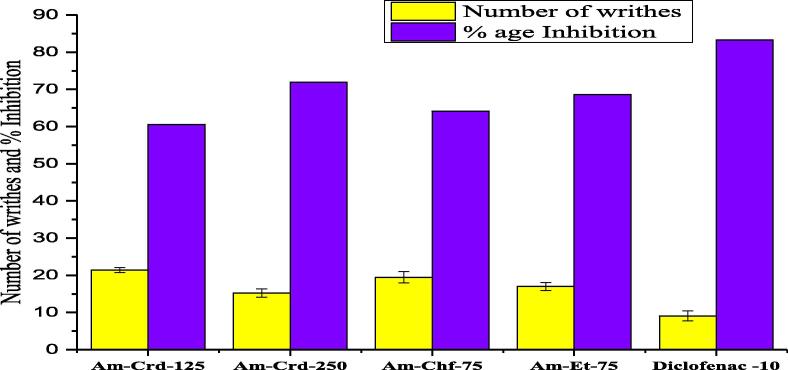
A. acid induced writhing model analgesic activity of *A. maritime*. **Key**: Am = *A. maritima,* Crd = Crude, Chf = Chloroform, Et = Ethyl acetate,

**Fig. 3:**
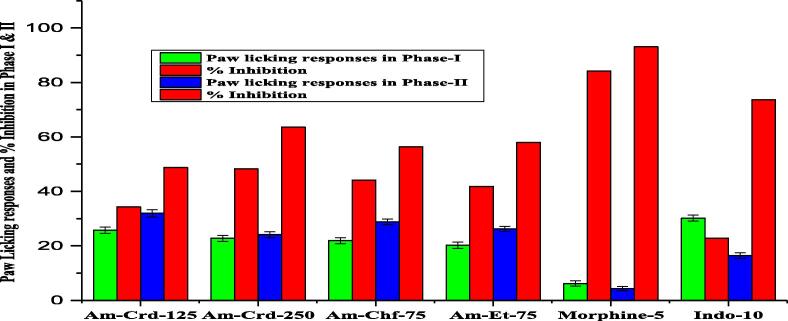
Formalin induced paw licking model analgesic activity of *A. maritima* of phase-I and Phase-II.

The results revealed a dose dependent pain relief and maximum inhibition (77.17 %, P < 0.001, n = 8) for the crude extract of *A. maritima* (Am- Crd) at 250 mg/kg as compared to control (Diclofenac sodium) at a dose of 10 mg/kg (88.42 %) (Fig. 2). Formalin test results of Phase-I and phase-IIrevealed dose-dependent inhibitory effectin mice with formalin induced nociception. The crude extracts (Am-Crd) at 250 mg/kg and ethyl acetate fractions at 70 mg/kgrevealed significantly higher inhibition of the neurogenic and inflammatory phases when compared with control (morphine 5 mg/kg and indomethacin 10 mg/kg) (Fig. 3).

#### 3.6.2 Hot plate test

**Fig. 4:**
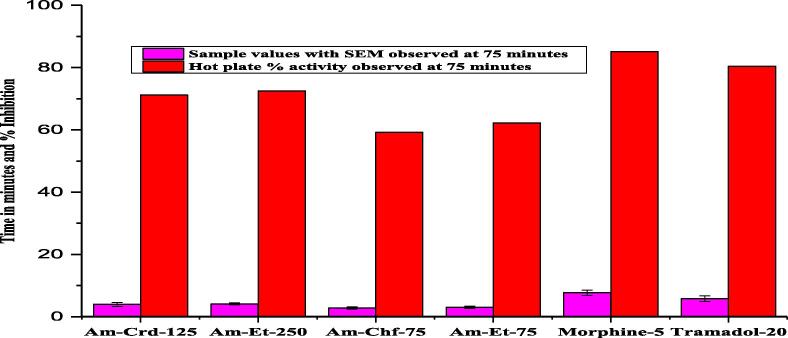
Hot plate tail flicking model analgesic activity of *A. maritima.*

The central analgesic effect of the samples is commonly assessed by Hot plate test. A significant inhibition was shown by crude extract (Am-Crd) at a dose of 250 and 125 mg/kg followed by fractions of ethyl acetate (Am-Et-75) and chloroform (Chf-75)as compared to the control group (tramadol and morphine)with the result of 80.45 % (P < 0.001, n = 8) and 85.12 % (P < 0.001, n = 8) (Fig. 4).

The crude extracts and fractions of chloroform and ethyl acetate exhibited an activity resembling more to that of morphine and tramadol, giving an idea towards the involvement of central mechanism. Therefore, in next step, a non-selective opioid antagonist (naloxone) was used in response of agonistic effects of morphine to find out the possible involvement of opioid receptor.

#### 3.6.3 Involvement of opioid receptor

The possible involvement of opioid receptor was determined using hot plate test. A reversal of inhibitory potential was observed in animals pre-treated with naloxone. The results show that naloxone (morphine and tramadol antagonist) showed no prominent effect. This agonistic and antagonistic effect of morphine/tramadol and naloxone indicates the absence of possible involvement of opioid receptors (Table S9).

#### 3.6.4 Involvement of ATP-sensitive K+ channel pathway

A possible role for ATP sensitive potassium channel in the analgesic effect of the plant extract and fractions was investigated. Glibenclamide pretreatment minutely modified the analgesic action of the tested samples. This observation suggests that ATP sensitive potassium channels are very partly involved in the analgesic action of the tested samples (Table S10).

## 4 Discussion

Medicinal plants faced issues related to adulteration of morphologically similar species (Ahmed et al., 2019). Genus *Artemisia* consist closely similar but morphologically different species which make *Artemisia* extremely difficult to identify correctly (Ghafoor 2002). The role of microscopy is highly important in the validation and identification of novel medicinal plants (Fatima et al., 2018). Pollen grains studies are extensively used in taxonomy for identification of different closely related flowering plants (Bahadur et al., 2019). The foliar epidermal featuresare used as an excellent diagnostic tool for the determination of taxonomic interaction in plants (Hussain et al., 2019). Microscopy of powder drugs helps in the authentication, identification and adulteration detection in the herbal drugs (Soni et al., 2011).The present microscopic studies of different parameters of *A. maritima* supported by previous studies such as;Hameed et al., 2020, Hussain et al., 2019, Ivashchenko and Ivanenko, 2017, Hayat et al., 2010. Working on the bark of different plants Singh et al., 2018, Xavier et al., 2015, Khan and Khan, 2020 reported similar organoleptic properties, tissues and cells. The current scheme of study is an easy step to distinguish unadulterated from adulterated ones.

From nutritional point of view value of fats fall <2 % because it is a macronutrient of less abundance. The protein level is usually 5 % or above and varies in different fruits (Demir and Özcan, 2001).In proximate analysis ash contents reveals the direct estimation of total quantity of minerals in a specific sample (Younis et al., 2016). The current study revealed that *A. maritima* contain sufficient nutrient which are in agreement with (Younis et al., 2016), Penuel et al., 2013, Sadia et al., 2014. The proper maintenance of physiological and biochemical functions of life need proper amount of minerals (Pontieri et al., 2014). Human health faces serious challenges and affecting globally due to the deficiency of minerals (Subhani et al., 2015).The current study revealed that *A. maritima* was found to contain higher concentration of Ca, Na, K and Zn. Heavy metals quantified in the current study did not exceededpermissible limits of WHO safety threshold.

According to Stanojević et al., 2009, Mustafa et al., 2010 methanolic fractions were found rich with total phenolic and total flavonoid contents similar results found in the current study.The quantity of phenolic contentsreported in *A. maritima*in the current study parallel to those reported by Pereira et al., 2018, Dib et al., 2017, Megdiche-Ksouri et al., 2015, Ghlissi et al., 2016, Al Jahid et al., 2016 with minor variations. Plants (herbs, vegetables, fruits) have a broad range of free radical scavenging constituents such as flavonoids, phenolic acids, tannins, alkaloids, terpenoids and other metabolites with high antioxidant activity (Krishnaiah et al., 2011). Different reports such as Pandey et al., 2017, Abiri et al., 2018, and Sharopov et al. (2020) confirmed the antioxidant status genus *Artemisa*. The antibacterial assays revealed that methanolic extracts and fractions of chloroform and ethyl acetate were found effective. The study such as Khan et al., 2014, Stappen et al., 2014 reported greater antimicrobial activity.The findings of cytotoxic effects of *A. maritima* supported byprevious work done of Pereira et al., 2018, Abiri et al., 2018. The highest antidiabetic activity supported by findings of Chen et al., 2019, Fatima et al., 2017. The *in-vitro* studies of the current studies provide details of different strains extracts and fractions which are different from the previous work done. Moreover they studied different species of *Artemisia*.These activities may be attributed to the active constituents present in *A. maritima*.

The abdominal constriction assay induced with acetic acid is helpful for finding out peripheral analgesic response (Neto et al., 2005). Formalin induced nociception is used to quantify the potentials of a substance to relieve continuous moderate pain produced (Oliveira et al., 2009). To find out the spinal pathways in the regulation of pain response the tail immersion test is used (Khatun et al., 2015).The opioid receptors are widely distributed in the peripheral and central nervous systems. In response to noxious stimuli the endogenous opiods activate the opioid receptors (Koneru et al., 2009). The cellular membrane glycoprotein are opioid receptors which are responsible to change the conduction of potassium (K^+^) and calcium (Ca^+^) ions (Du Pen et al., 2004). The study outcomes for possible mechanism of painare in agreement with the previous findings of Habib and Waheed (2013), (Afsar et al., 2013) and (Qing-Hu et al., 2015).Previous work done on different *Artemisia* species for analgesic response also reported dose dependent and solvent based activities such as; Hadi et al., 2014, Habib and Waheed, 2013, Ashok and Upadhyaya, 2013. The analgesic activities in the current study confirmed the traditional uses of genus *Artemisia* administered as pain with scientific basisand confirmed the existence of antinociceptive potentials of *A.maritima*.

According to Singh et al. (2010), spectroscopic techniques can be used in quality control of herbal medicines.The findings of current study for FTIR are supported by previous studies such as Kim et al., (2009). The compounds quantified and reported in GC–MS and HPLC analysis (pyrogallol and ellagic acid in high quantity) confirmed its active biological natureas antioxidant, antimicrobial, cytotoxic, antidiabetic and analgesicfrom the literature such as Cynthia et al., 2018, Sampath et al., 2021, Mondal et al., 2020, Zheng et al., 2018, Savic et al., 2019, Gupta et al., 2019, Fatima et al., 2017, Chen et al., 2018. Therefore, it is concluded that the presence of these compounds are responsible for the potentmedicinal nature of the *A. maritima*and can be attributed to biological activities revealed by *A.maritima* in the current study.

## 5 Conclusion

Genus *Artemisia* is highly medicinal and well known for its medicinal and pharmacological value. The species of genus *Artemisia* are closely related therefore proper authentication and identification is highly important. This study was therefore focused on the microscopic characterization which revealed useful information for identification of the selected plant. Biological evaluation (*In-vitro* and *In-vivo*) revealed good results which concluded that *A. maritima* has an excellent potential to treat the diseases related to our performed experiments. These results also confirmed the ethno pharmacological uses of this species for different ailments. The phytochemical characterization provided useful information about the active constituents and biomolecules found in *A. maritima* which may be attributed to the observed biological potential. However, further experiments in this connection are needed to confirm the observed biological potential and also to isolate responsible compounds in pure state.

## Declaration of Competing Interest

The authors declare that they have no known competing financial interests or personal relationships that could have appeared to influence the work reported in this paper.
