# Supplementary material for: Pharmacognostic evaluation of Artemisia maritima L. a highly medicinal specie of genus Artemisia
Source: Saudi J Biol Sci. 2022 Aug 17;29(10):103419. doi: 10.1016/j.sjbs.2022.103419 (PMC9434231; doi:10.1016/j.sjbs.2022.103419)
Supplement: Supplementary data 1 [file mmc1.docx]

Supplementary file

**PHARMACOGNOSTIC EVALUATION OF *ARTEMISIA MARITIMA L.* A HIGHLY MEDICNAL SPECIE OF GENUS *ARTEMISIA***

**
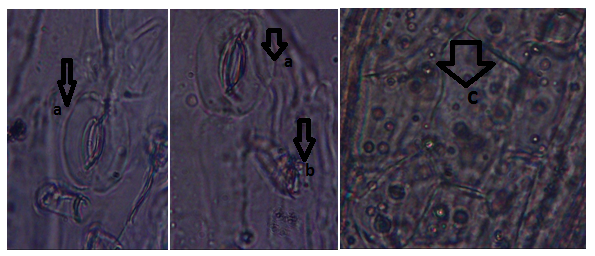
**

**Figure S1:** Foliarlight microscopic observations of *A. maritima* showing (a) anisocytic stomatal complex (b) trichome (c) elongated rectangular shape, smooth wall margins epidermal cells

**
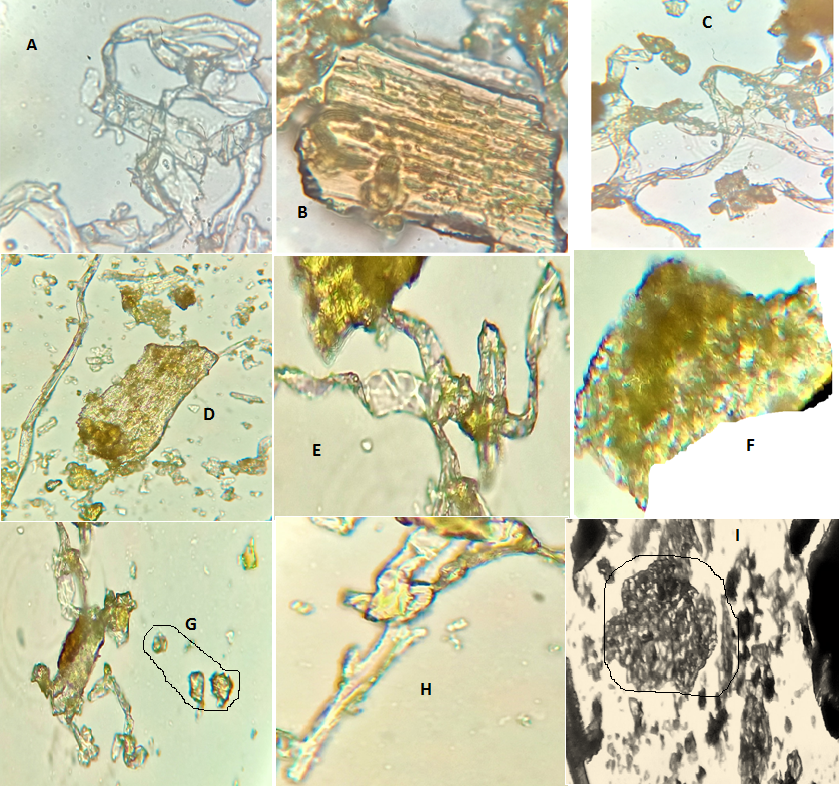

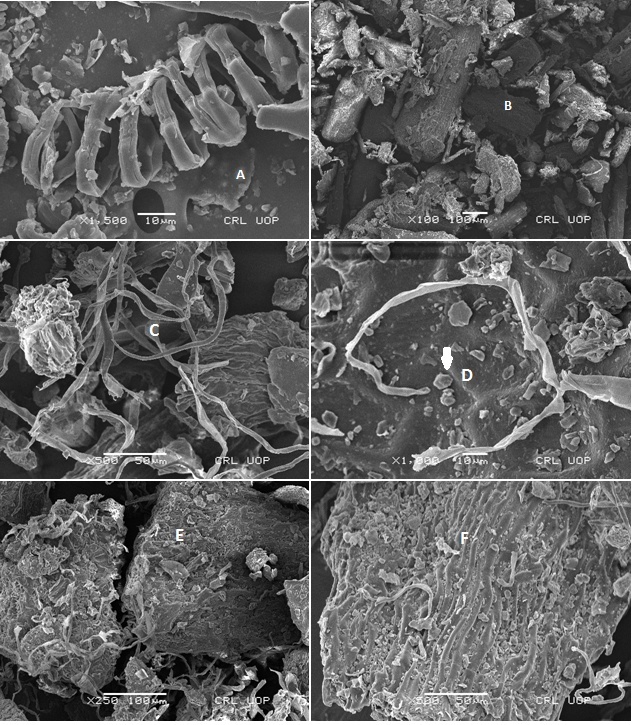
**

**Figure S2: LM** (A) xylem fibers, (B) xylem vessels, (C) long single phloem fibers **SEM:** (A) spiral xylem vessels, (B) phloem fibers, (C) xylem fibers, (D) prismatic calcium oxalate crystals

**Figure S3:** Percentage of nutritional contents (grams per 100 gram dry weight) in *A. maritima*

**Figure S4:** Elemental analysis for *A. maritima.*

**
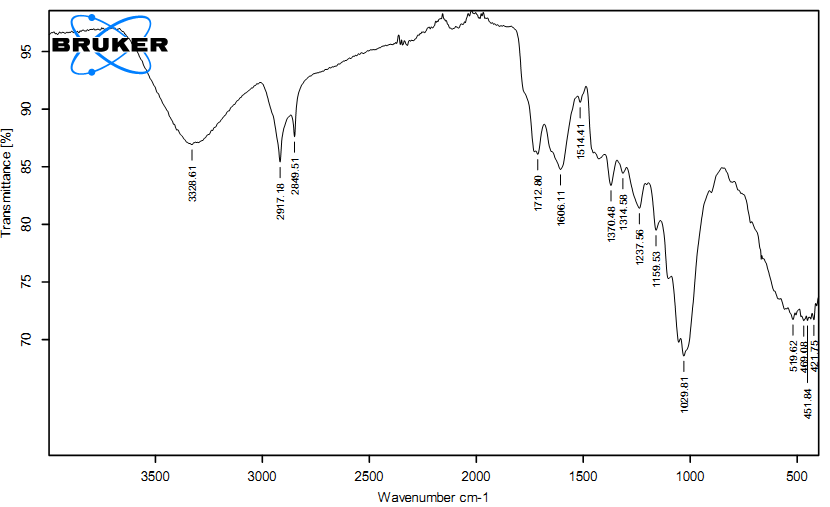
**

**Figure S5:** FTIR chromatogram identifying corresponding functional groups in *A*.*maritima*.


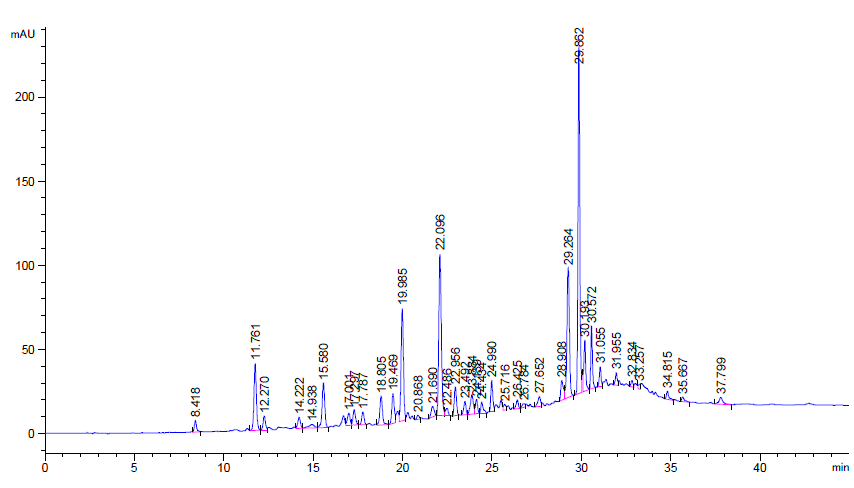


**Figure S6:** HPLC-UV chromatogram of *A. maritima* methanolic extract

**Figure S7:** GC-MS chromatogram of *A. maritima* methanolic extract


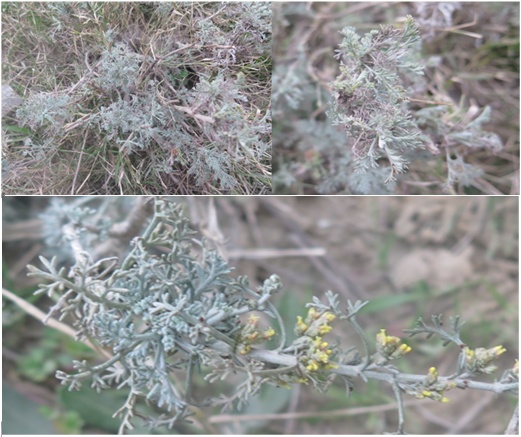


**Plate 1:** *A. maritima* in its natural habitats with its vegetative parts (leaves) and floral parts (inflorescence) collected from Kanju Township lower Swat with voucher number ICP090618.

**
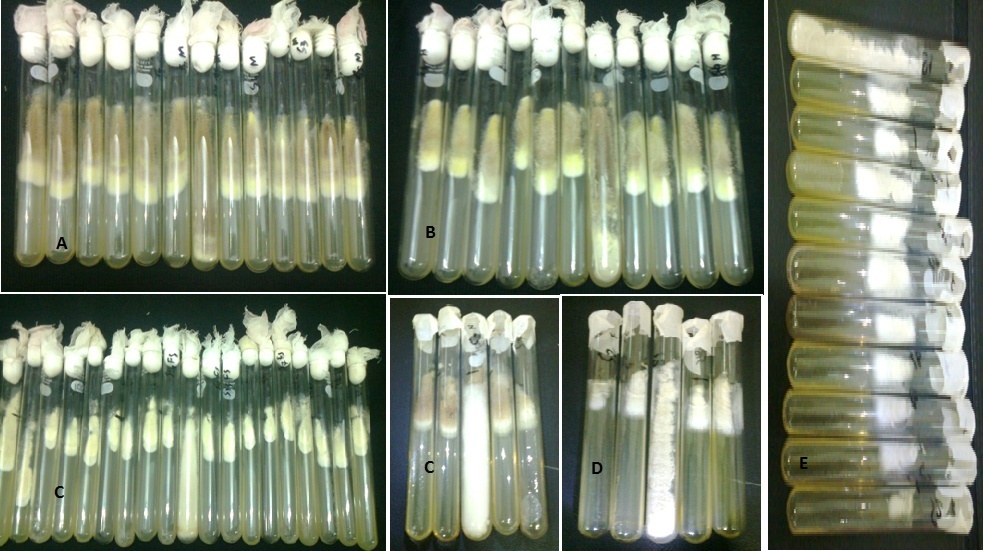
**

**Plate 2:** Antifungal activities based on tube dilution method


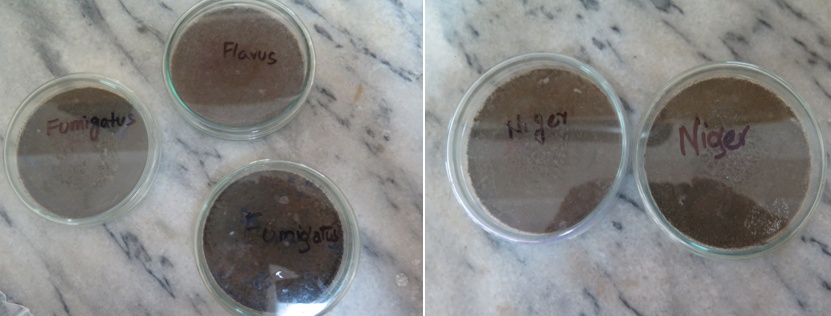


**Plate 3:** Antifungal activities based on disc diffusion method

**
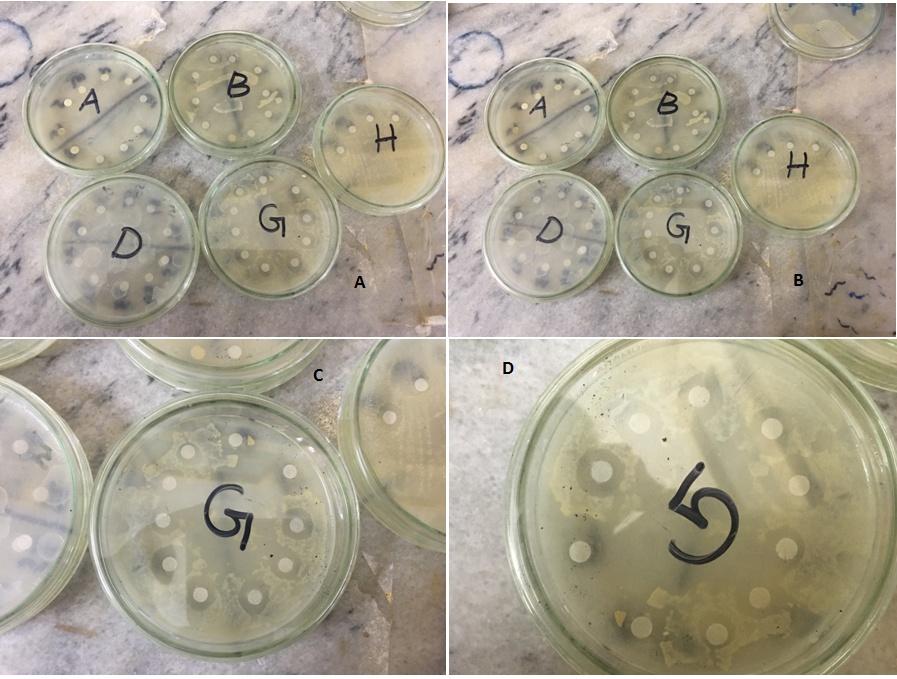
**

**Plate 4:** Antibacterial activities based on disc diffusion method

**Table S1:** LM and SEM evaluation of *A. maritima*for different powder characters

| **A. maritima** | **Xylem Vessels** | | **Phloem sieve elements** | | **Phloem fibers** | | **Cork cells** | |
| --- | --- | --- | --- | --- | --- | --- | --- | --- |
|  | **Length** | **Width** | **Length** | **Width** | **Length** | **Width** | **Length** | **Width** |
| **LM** | 30.1±0.18 | 15.4±0.28 | 33.2±0.31 | 17.7±0.92 | 14.3±0.29 | 8.1±0.17 | 9.6±0.47 | 5.1±0.32 |
| **SEM** | 15.2±0.12 | 7.2±0.15 | 19.5±0.08 | 9.9±0.03 | 8.7±0.05 | 5.8±0.07 | 6.5±0.05 | 3.9±0.06 |

LM = Light microscopy, SEM = scanning electron microscopy (Mean ± SEM (n=5 number)

**Table S2:** Qualitative and quantitative estimation of phytochemical in *A.maritima*

| **Phytochemicals** | **Qualitative** | **Quantitative** |
| --- | --- | --- |
| Saponin | ++ |  |
| Terpenes | ++ |  |
| Alkaloids Dragendroff’s test | ++ |  |
| Alkaloids Mayer’s test | ++ |  |
| Tannins | + |  |
| Flavonoids | ++ | 7.67±0.069 |
| Phenolics | ++ | 17.64±0.574 |

**Table S3:** Identification of different functional groups from FTIR analysis of *A. maritima.*

| **Functional Groups/Biomolecules** | **Corresponding range** |
| --- | --- |
| Alcohols, Phenols, Amines, Amides (Stretch), Carboxylic acid | 3400-3300 |
| Alkanes, Aldehyde, Carboxylic acid, Stretching C-H | 2950-2900 |
| Alkanes, Aldehyde, Carboxylic acid, Stretching C-H | 2900-2800 |
| Carboxylic acid, Ketone | 1800-1700 |
| Alkene, Amines and Amides (Stretch), Adenine vibration in DNA | 1700-1600 |
| Aromatic, Nitro (R-NO2) Stretch, (C=C)-diagnostic for the presence of a carotenoid | 1600-1500 |
| Alkanes (bend), Nitro (R-NO2) Stretch | 1500-1400 |
| Fluoride, Sulfones, Sulfonyl Chlorides, Sulfates, Sulfonamides, Stretching C-O,Deformation N-H, C-H | 1400-1350 |
| Fluoride, Sulfones, Sulfonyl Chlorides, Sulfates, Sulfonamides | 1350-1300 |
| Amines, Fluoride,Alcohols, Ethers, Esters | 1250-1225 |
| Amines, Fluoride,Alcohols, Ethers, Esters, C-O of proteins and carbohydrates | 1200-1150 |
| Amines, Fluoride,Alcohols, Ethers, Esters | 1150-1100 |
| Sulphoxide,Amines, Fluoride,Alcohols, Ethers, Esters | 1100-1050 |
| Amines, Fluoride,Alcohols, Ethers, Esters | 1050-1000 |
| Aromatic (Bend), Glycogen,Carbohydrate residues | 1000-900 |
| Alkenes (Bend), Bromide iodide, Chloride, Glycogen, Carbohydrate residues | 700-650 |
| Alkyl halide (Stretch), Glycogen, Carbohydrate residues | 600-550 |
| Alkyl halide, Carbohydrate residues | 550-500 |
| Disulphide, Carbohydrate residues | 550-500 |
| Protein amide, Oligo sacharide, Glycogen, Carbohydrate rsidue | 450-400 |

**Table S4:** Compounds identified from GC-MS analysis in *A. maritima*

| **S.NO** | **Area %** | **RT** | **Compounds** | **Formula** | **Mol weight** |
| --- | --- | --- | --- | --- | --- |
| 1 | 0.04 | 2.47 | .psi.,.psi.-Carotene,3,4-didehydro-1,2-dihydro-1-methoxy- | C41H58O | 566 |
| 2 | 2.02 | 4.46 | Eucalyptol | C10H18O | 154 |
| 3 | 0.11 | 4.99 | N-[4-(4-Chlorophenyl)isothiazol-5-yl)-1-methylpiperidin-2-imine | C18H35NO | 281 |
| 4 | 1.19 | 6.36 | Camphor | C10H16O | 152 |
| 5 | 6.02 | 10.82 | Caryophyllene | C15H24 | 204 |
| 6 | 1.69 | 13.26 | Caryophyllene oxide | C15H24O | 220 |
| 7 | 0.53 | 14.71 | Cholestan-3-ol, 2-methylene-, (3á,5à)- | C28H48O | 400 |
| 8 | 0.29 | 15.4 | 4a-Hydroxy-4-nitroperhydronaphthalen-1-one | C18H34O2 | 282 |
| 9 | 0.85 | 15.81 | 7-Methyl-Z-tetradecen-1-ol acetate | C17H32O2 | 268 |
| 10 | 0.93 | 17.17 | Neoisolongifolene, 8,9-epoxy- | C15H22O | 218 |
| 11 | 0.15 | 18.62 | Dasycarpidan-1-methanol, acetate(ester) | 20H26N2O2 | 326 |
| 12 | 22.27 | 19.17 | Hanphyllin | C15H20O3 | 248 |
| 13 | 16.35 | 20.68 | Anobin | C15H20O5 | 280 |
| 14 | 3.07 | 25.67 | Rhodopin | C40H58O | 554 |
| 15 | 3.1 | 25.93 | á-Sitosterol | C29H50O | 414 |

**Table S5:** MIC values of antifungal activities of *A. maritima* (using disk diffusion method)

| **Disc diffusion method** | **Fractions** | **Fungal strains** | | | | | | |
| --- | --- | --- | --- | --- | --- | --- | --- | --- |
|  |  | ***A. niger*** | ***A. flavus*** | ***A. fumigates*** | ***Mucor sp.*** | ***H. solani*** | ***C. albicans*** | ***F. solani*** |
|  |  |  |  |  |  |  |  |  |
|  |  | **MIC (mg/ml)** | **MIC (mg/ml)** | **MIC (mg/ml)** | **MIC (mg/ml)** | **MIC (mg/ml)** | **MIC (mg/ml)** | **MIC (mg/ml)** |
|  | **Meth** | 15 | - | 15 | 10 | 10 | 10 | 10 |
|  | **CHl** | 10 | - | 15 | 10 | 15 | 10 | 10 |
|  | **E.a** | 15 | 10 | 15 | 10 | 15 | 15 | 10 |

**Table S6:** Results of MIC values recorded from antibacterial activities of different fractions of *A. maritima*

| **Minimum inhibition concentration/MIC (mg/ml)** | | | | |
| --- | --- | --- | --- | --- |
| **Fractions** | ***B. subtilis*** | ***S. aureus*** | ***E. coli*** | ***P. aeruginosa*** |
|  | **mg/ml** | **mg/ml** | **mg/ml** | **mg/ml** |
| **Meth** | 3.75 | 1.87 | 1.87 | 1.87 |
| **CHl** | - | - | - | 3.75 |
| **E.a** | - | - | - | - |

**Table S7**: Brine shrimps (*Artemia salina*), cytotoxic activity of methanolic extracts of *A. maritima*

| **Conc. (µg/ml)** | **No. of live** | **No. of dead** | **Percent activity/death** | **Net % activity/death** | **LC_50_** |
| --- | --- | --- | --- | --- | --- |
|  |  |  |  |  |  |
| 50 | 4 | 6 | 60 | 40 | 20 µg/ml |
| 100 | 3 | 7 | 70 | 50 |  |
| 200 | 0 | 10 | 100 | 80 |  |

**Table S8**: Anti-diabetic activity expressed in % of inhibition against α- amylase

| **S.No** | **Fractions** | **Absorption** | **ODx-ODy** | **% Inhibition** | **Control** | **Absorption** |
| --- | --- | --- | --- | --- | --- | --- |
| 1 | Methanolic | 1.261 | 0.251 | 12.61^a^ | Positive | 1.65 |
| 2 | Chloroform | 1.213 | 0.203 | 10.20^b^ | Negative | 1.01 |
| 3 | Ethyl acetate | 1.16 | 0.15 | 7.53^c^ | Blank | 3 |

**Table S9:** Opioid antagonist (naloxone) and agonestic (tramadol) effects by hot plate method

| **Treat/Dose (mg)** | **Time intervals in minutes** | | | | | | | |
| --- | --- | --- | --- | --- | --- | --- | --- | --- |
|  | **0** | **15** | **10** | **45** | **60** | **75** | **90** | **120** |
| **Control (2% Tween 80)** | 1.11±0.35 | 1.09±0.51 | 1.15±0.81 | 1.11±0.72 | 1.12±0.51 | 1.01±0.54 | 1.08±0.91 | 1.09±0.73 |
| **Am-Crd-125** | 1.02±0.51 | 1.01±0.59 | 1.07±0.69 | 1.17±0.61 | 1.07±0.39 | 1.08±0.91 | 1.01±0.63 | 1.02±0.69 |
| **Am-Crd-250** | 1.05±0.71 | 1.15±0.61 | 1.18±0.91 | 1.10±0.57 | 1.11±0.92 | 1.01±0.44 | 1.17±0.70 | 1.08±0.61 |
| **Am-Chf-75** | 1.01±0.35 | 1.11±0.72 | 1.10±0.61 | 1.11±0.61 | 1.01±0.72 | 1.01±0.61 | 1.11±0.87 | 1.18±0.72 |
| **Am-Et-75** | 1.07±0.61 | 1.15±0.61 | 1.01±0.51 | 1.16±0.91 | 1.08±0.91 | 1.17±0.67 | 1.01±0.52 | 1.07±0.49 |
| **Tra+Nal (+ve)** | 1.29±1.11 | 1.11±1.03 | 1.71±1.02 | 1.14±1.01 | 1.18±0.87 | 1.17±0.89 | 1.09±0.70 | 1.11±0.52 |

**Table S10:** ATP sensitive potassium channel pathway using A. acid induced writhing model

| **Treatment/Dose** | **Writhes** | **% inh** |
| --- | --- | --- |
| **Control (2% Tween 80)** | 54.22±1.29 |  |
| **Am-Crd-125** | 25.38±1.55 | 53.18 |
| **Am-Crd-250** | 21.13±1.17 | 61.02 |
| **Am-Chf-75** | 23.18±1.43 | 57.24 |
| **Am-Et-75** | 22.73±1.10 | 58.07 |
| **Glibenclamide** | 45.47±1.25 | 16.12 |
